# Transformers-sklearn: a toolkit for medical language understanding with *transformer-based* models

**DOI:** 10.1186/s12911-021-01459-0

**Published:** 2021-07-30

**Authors:** Feihong Yang, Xuwen Wang, Hetong Ma, Jiao Li

**Affiliations:** Institute of Medical Information and Library, Chinese Academy of Medical Sciences/Peking Union Medical College, 3rd Yabao Road, Beijing, 100020 China

**Keywords:** Transformer, NLP, Toolkit, Deep Learning, Medical Language Understanding

## Abstract

**Background:**

*Transformer* is an attention-based architecture proven the state-of-the-art model in natural language processing (NLP). To reduce the difficulty of beginning to use *transformer-based* models in medical language understanding and expand the capability of the *scikit-learn* toolkit in deep learning, we proposed an easy to learn Python toolkit named *transformers-sklearn.* By wrapping the interfaces of *transformers* in only three functions (i.e., fit, score, and predict), *transformers-sklearn* combines the advantages of the *transformers* and *scikit-learn* toolkits.

**Methods:**

In *transformers-sklearn*, three Python classes were implemented, namely, *BERTologyClassifier* for the classification task, *BERTologyNERClassifier* for the named entity recognition (NER) task, and *BERTologyRegressor* for the regression task. Each class contains three methods, i.e., *fit* for fine-tuning *transformer-based* models with the training dataset, *score* for evaluating the performance of the fine-tuned model, and *predict* for predicting the labels of the test dataset. *transformers-sklearn* is a user-friendly toolkit that (1) Is customizable via a few parameters (e.g., *model_name_or_path* and *model_type*), (2) Supports multilingual NLP tasks, and (3) Requires less coding. The input data format is automatically generated by *transformers-sklearn* with the annotated corpus. Newcomers only need to prepare the dataset. The model framework and training methods are predefined in *transformers-sklearn*.

**Results:**

We collected four open-source medical language datasets, including *TrialClassification* for Chinese medical trial text multi label classification, *BC5CDR* for English biomedical text name entity recognition, *DiabetesNER* for Chinese diabetes entity recognition and *BIOSSES* for English biomedical sentence similarity estimation.

In the four medical NLP tasks, the average code size of our script is 45 lines/task, which is one-sixth the size of *transformers*’ script. The experimental results show that *transformers-sklearn* based on pretrained BERT models achieved macro F1 scores of 0.8225, 0.8703 and 0.6908, respectively, on the *TrialClassification*, *BC5CDR* and *DiabetesNER* tasks and a *Pearson correlation* of 0.8260 on the *BIOSSES* task, which is consistent with the results of *transformers*.

**Conclusions:**

The proposed toolkit could help newcomers address medical language understanding tasks using the *scikit-learn* coding style easily. The code and tutorials of *transformers-sklearn* are available at https://doi.org/10.5281/zenodo.4453803. In future, more medical language understanding tasks will be supported to improve the applications of *transformers_sklearn*.

## Background

*Transformer* is an attention-based architecture proposed by Vaswani et al. [1], which has been proved to be the state-of-the-art model by BERT [2] (i.e., Bidirectional Encoder Representations from Transformers), RoBERTa [3] (i.e., a Robustly Optimized BERT pre-training Approach), etc. With the development of natural language processing (NLP) technology, *transformer*-based models have emerged. To effectively utilize these models and evaluate their performance in downstream tasks, a Python library of *transformer*-based models, namely, *transformers* [4], has been developed by gathering state-of-the-art general purpose pre-trained models under a unified application program interface (API) together with an ecosystem of libraries. *transformers* has been reported to have been used in more than 200 research papers and included either as a dependency or with a wrapper in several popular NLP frameworks such as AllenNLP [5] and Flair [6].

*scikit-learn* [7], which is a Python module integrating a wide range of state-of-the-art machine learning algorithms for medium-scale supervised and unsupervised problems, is one of the most popular machine-learning toolkits. It is friendly to newcomers to apply it in machine learning tasks. Based on *scikit-learn*, Lemaitre G et al. proposed *imbalanced-learn* [8] to provide various methods to cope with the imbalanced dataset problem frequently encountered in machine learning and pattern recognition. Szymański P and Kajdanowicz T developed a Python library named *scikit-multilearn* [9] for performing multi label classification. Löning M et al. present *sktime* [10], which is a *scikit-learn* compatible the Python library with a unified interface for machine learning with time series. De Vazelhes W et al. implemented supervised and weakly supervised distance metric learning algorithms and wrapped them in a Python package named *metric-learn* [11]. These works made *scikit-learn* more powerful and efficient in specific domain tasks.

As known, the *transformers* toolkit is well designed and friendly to professional researchers and engineers. However, for newcomers who have no knowledge of *transformers*, it is still time-consuming to learn the background knowledge about *transformers* from scratch. *scikit-learn* is designed to make machine learning for easy use, but there is still a gap between machine learning and deep learning algorithms in *scikit-learn*.

To reduce the difficulty of getting started with *transformer-based* models and expand the capability of *scikit-learn* in deep learning, we combine the advantages of the *transformers* and *scikit-learn* toolkits and propose a Python toolkit named *transformers-sklearn*. The proposed toolkit aims to make *transformer-based* models convenient for beginners by wrapping the interfaces of *transformers* in only three APIs (i.e., fit, score, and predict). With *transformers-sklearn*, newcomers could use *transformer-based* models to address their NLP tasks, even though they had no previous knowledge of *transformer*. The users can pay more attention on the NLP task itself, with preparing the training dataset for fitting, the development dataset for scoring the model, and the test dataset for predicting.

The primary contributions of this paper are as follows. (1) We proposed transformers-sklearn, which makes transformer-based models for easy use and expands the capability of scikit-learn in deep learning methods. (2) To validate the performance of transformers-sklearn, experiments were conducted on four NLP tasks based on English and Chinese medical language datasets. We also compared transformers-sklearn with the widely used NLP toolkits such as *transformers* and *UER* [12]. (3) The code and tutorials of transformers-sklearn are available at https://doi.org/10.5281/zenodo.4453803.

## Methods

In *transformers-sklearn*, there are three Python classes designed for classification, named entity recognition (NER), and regression tasks. Each class contains three methods, namely, *fit*, *score*, and *predict*.

### Python classes

*transformers-sklearn* was implemented with three Python classes, which are *BERTologyClassifier* for the classification task, *BERTologyNERClassifier* for the named entity recognition (NER) task, and *BERTologyRegressor* for the regression task. *BERTologyClassifier* and *BERTologyNERClassifier* are subclasses of *BaseEstimator* and *ClassifierMixin* implemented by the *scikit-learn* toolkit. *BERTologyRegressor* is the subclass of *BaseEstimator* and *RegressorMixin* implemented by *scikit-learn*.

All classes could be customized by setting the values of multiple parameters. Among these parameters, *model_type* is used to specify which type of model initialization style should be used, and *model_name_or_path* is used to specify which pre-trained model should be used. There are six model initialization types, namely, BERT, RoBERTa, XLNet [13], XLM [14], DistilBERT [15] and ALBERT [16]. All these models are implemented based on a *transformer*, but they differ in their data processing. More details about the parameters are shown in Table 1.

**Table 1:** The common parameters of the Python classes in *transformers-sklearn*

Name	Function
*model_type*	Specifies which type of model initialization style should be used
*model_name_or_path*	Specifies which pre-trained model should be used
*max_seq_length*	Sets the max length of the sequence that could be accepted
*per_gpu_train_batch_size*	Sets the batch size per GPU
*learning_rate*	Sets the learning rate of the model
*num_train_epochs*	Sets the number of training epochs of the model
*no_cuda*	Sets whether the GPU is used for training or predicting

### Class methods

The same as with the class methods of *scikit-learn*, three methods (i.e., *fit*, *score,* and *predict*) were implemented in each Python class of *transformers-sklearn*. The *fit* and *score* methods accept two parameters, which are *X* and *y*. *X* is a container of sentences or documents, and *y* contains the corresponding labels. *X* and *y* could be one of the following Python data types: *list*, *ndarray* implemented by *numpy* [17], and *DataFrame* implemented by *panda*s [18]. The *predict* method only requires parameter *X*.

The functions of the above class methods were as follows:

1. *Fit*. This method was used to fine-tune the customized pre-trained model following the configuration of the parameters in each class (i.e., *BERTologyClassifier* or *BERTologyNERClassifier* or *BERTologyRegressor*). In this method, the training set was automatically transformed to the specific format and then fed into the customized *transformer-based* model for fine-tuning.
2. *Score*. This method was used to evaluate the performance of the fine-tuned model. For example, in the classification task, this method would return the common evaluation indexes such as the precision, recall and F1-score for each type of label.
3. *Predict*. This method was used to predict the labels of a given dataset.

Traditionally, it is difficult for newcomers to address their NLP problems using the *transformers*-based methods. For instant, a user would like to apply the BERT model to address a binary classification task, thereafter, four steps were needed to fine-tune the pre-trained BERT model as follows:

1. **Data preparation**. The training set is transformed to a special format for the BERT model. The user needs to learn about the data processing of BERT.
2. **Model configuration**. The user customizes the model with fully understanding the architecture of BERT.
3. **Fine-tuning model**. The user determines epochs that are used to fine-tune the customized BERT.
4. **Saving fine-tuned model**. The user saves the fine-tuned model to the target path.

The four steps mentioned above increased the developmental difficulty for newcomers, and it is time-consuming for them to learn the necessary background knowledge. In our work *transformers-sklearn*, the four steps are implemented automatically in the *fit* method and transparent to users.

### Workflow

**Fig. 1:**
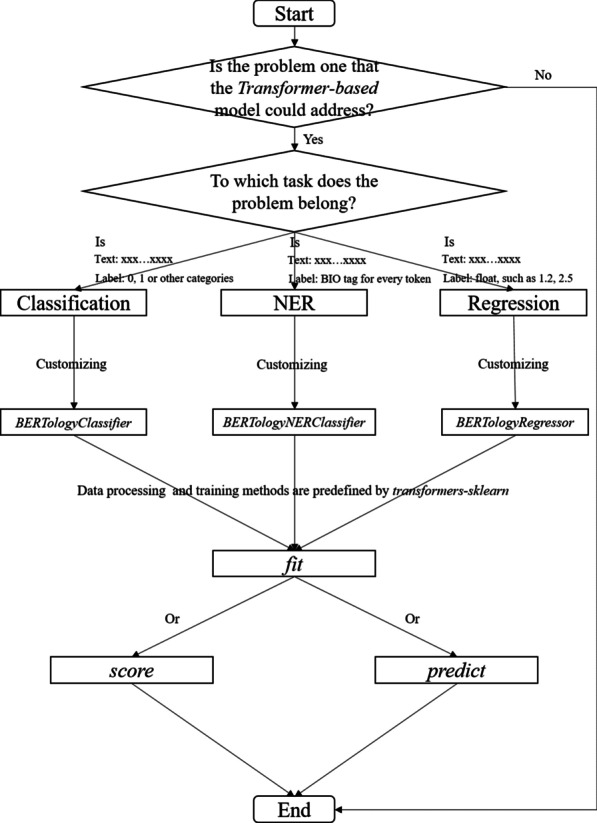
Workflow of using *transformers-sklearn* to address NLP problems

As shown in Fig. 1, when facing an NLP task, the user first determines whether the *transformer-based* models could address the problem. If the so, the user should choose one class from *BERTologyClassifier*, *BERTologyNERClassifier* and *BERTologyRegressor*, which could be customized by setting the parameters, depending on to which class the problem belongs. After customizing the chosen class, the user feeds the datasets into the *fit* method. Using the NER task as an example, the input data format is defined as Table 2. As shown the *text* field contains segmented texts to be labelled, and the *label* field contains the corresponding medical named entities obtained by manual annotations. Then, *transformers-sklearn* would conduct the fine-tuning process automatically. Finally, the user could evaluate the fine-tuned model using the *score* method or deploy the fine-tuned model in practice using the *predict* method. During the whole workflow, it is possible for the user to dispense with understanding the internal mechanisms of the chosen *transformer-based* model.

**Table 2:** An example of the NER input data format in the *BERTologyNERClassifier*

Input data field	Example
Text	[…[“Naloxone”, “reverses”, “the”, “antihypertensive”, “effect”, “of”, “clonidine”, “.”], …]
Label	[…[“B-Chem”, “O”, “O”, “O”, “O”, “O”, “B-Chem”,”O”], …]

## Experiments

We conducted comparison experiments to validate the effectiveness of *transformers-sklearn* on multilingual medical NLP tasks. We selected three popular transformer-based model types from our package, i.e., BERT, RoBERTa, and ALBERT, and compared them with the original *transformers* and *UER* [12]. The pre-trained models of different model types can be downloaded automatically or manually from the community [19], as shown in Table 3. All experiments were conducted on four Tesla V100 16 GB GPUs with the initial number of training epochs set to 3, the learning rate set to 5e-5 and the other parameters set to their default values. The parameters such as the epochs and learning rate can be adjusted manually according to specific experiments.

**Table 3:** Pre-trained models and URLs

Model	URL
*bert-base-chinese*	https://huggingface.co/bert-base-chinese
*bert-base-cased*	https://huggingface.co/bert-base-cased
*chinese-roberta-wwm-ext*	https://huggingface.co/hfl/chinese-roberta-wwm-ext
*roberta-base*	https://huggingface.co/roberta-base
*albert_chinese_base*	https://huggingface.co/voidful/albert_chinese_base
*albert-base-v2*	https://huggingface.co/albert-base-v2

### Corpus

To assess the performance of *transformers-sklearn* on medical language understanding, we collected the following four English and Chinese medical datasets (*TrialClassification*, *BC5CDR, DiabetesNER, and BIOSSES*) from the NLP community as our experimental corpora. More details on the four datasets can be found in Table 4.

1. *TrialClassification *[20]*.* This dataset contains 38,341 Chinese clinical trial sentences and is labelled with 45 classes. It was developed for Chinese medical trial text multilabel classification.
2. *BC5CDR *[21]*.* This dataset is a collection of 1,500 PubMed titles and abstracts selected from the CTD-Pfizer corpus and was used in the BioCreative V chemical-disease relation task. It was developed for English biomedical text name entity recognition.
3. *DiabetesNER *[22]. The dataset contains more than 9,556 Chinese medical named entity identification samples. It was developed for Chinese diabetes entity recognition. We randomly selected 80% of the data for training and 20% of the data for testing.
4. *BIOSSES *[23]*.* This dataset is a corpus of 100 sentence pairs selected from the Biomedical Summarization Track Training Dataset in the biomedical domain. It was collected for English biomedical sentence similarity estimation. Here, we randomly selected 80% of the data for training and 20% of the data for testing.

**Table 4:** The open-source datasets of the four English and Chinese Medical NLP tasks

Name	NLP Task	Language	Domain	Metric
TrialClassification [20]	Classification	Chinese	Clinical Trial	*Macro F1*
BC5CDR [21]	NER	English	PubMed titles and abstracts	*Macro F1*
DiabetesNER [22]	NER	Chinese	Diabetes Papers	*Macro F1*
BIOSSES [23]	Regression	English	Biomedical	*Pearson correlation*

### Evaluation

Two types of evaluation indexes were used for scoring, which are the macro F1 and Pearson/Spearman correlation*.* For the macro F1, set *n* classes as *C*_*1*_, *C*_*2*_, … *C*_*n*_. The precision for each class was defined as *P*_*i*_, which equals the number of correct predictions *C*_*i*_ divided by the number of prediction *Ci*. The recall for each class was defined as *R*_*i*_, which equals the number of correct predictions *C*_*i*_ divided by the number of predictions *Ci*. Then, the macro F1 score of the tasks were calculated as follows:

1 $${\text{Macro}}\,{\text{F}}1 = \left( \frac{1}{n} \right)\mathop \sum \limits_{i = 1}^{n} \frac{{2 \times P_{i} \times R_{i} }}{{P_{i} + R_{i} }}$$

For the Pearson correlation, set *y* as the true value of given dataset and *y_pred* as the value predicted by the model. Then, the Pearson correlation was calculated as follows:

2 $$\rho_{y,y\_pred} = \frac{{E\left( {yy_{pred} } \right) - E\left( y \right)E\left( {y\_pred} \right)}}{{\sqrt {E\left( {y^{2} } \right) - E^{2} \left( y \right)} \sqrt {E\left( {y_{pred}^{2} } \right) - E^{2} \left( {y\_pred} \right)} }}$$

## Results

The performances of the BERT model implemented by *transformers-sklearn*, *transformers* and *UER* in the four medical NLP tasks are shown in Table 5. The *transformers-sklearn* toolkit achieved macro F1 scores of 0.8225, 0.8703 and 0.6908 in the *TrialClassification*, *BC5CDR* and *DiabetesNER* tasks, respectively, and a Pearson correlation of 0.8260 in the *BIOSSES* task, which are consistent with the results of *transformers*.

**Table 5:** The experimental results of *transformers-sklearn*, *transformers* and *UER* in four medical NLP tasks (mode_type = “bert”)

Name	Score			Second			Lines of code			Pre-trained model
	Ours	*Transformers*	UER	Ours	Transformers	UER	Ours	*Transformers*	UER	
TrialClassification	0.8225^a^	**0.8312**^a^	0.8213^a^	1198	1227	764	38	246	412	*bert-base-chinese*
BC5CDR	**0.8703**^a^	0.8635^a^	-	471	499	-	41	309	-	*bert-base-cased*
DiabetesNER	0.6908^a^	0.6962^a^	**0.7166**^a^	1254	1548	2805	63	309	372	*bert-base-chinese*
BIOSSES	**0.8260**^b^	0.8200^b^	-	19	15	-	41	246	-	*bert-base-cased*

^a^The value of *Macro F1, where the bolded one indicates the best performance.*

^b^The value of *Person correlation, where the bolded one indicates the best performance.*

Tables 6 and 7 show the performances of the RoBERTa and ALBERT models, respectively. The RoBERTa model in *transformers-sklearn* achieved macro F1 scores of 0.8148, 0.8528, and 0.7068 in the *TrialClassification*, *BC5CDR* and *DiabetesNER* tasks, respectively, and a Pearson correlation of 0.39962 in the *BIOSSES* task. The ALBERT model in *transformers-sklearn* achieved macro F1 scores of 0.7142, 0.8422, and 0.6196 in the three respective tasks and a Pearson correlation of 0.1892 in the *BIOSSES* task.

**Table 6:** The experimental results of *transformers-sklearn* and *transformers* in four medical NLP tasks (mode_type = “roberta”)

Name	Score		Second		Lines of code		Pre-trained model
	Ours	*Transformers*	Ours	Transformers	Ours	*Transformers*	
TrialClassification	0.8148^**a**^	**0.8231**^a^	1206	1208	38	246	*chinese-roberta-wwm-ext*
BC5CDR	**0.8528**^**a**^	0.8461^a^	460	504	41	309	*roberta-base*
DiabetesNER	0.7068^a^	**0.7184**^a^	1445	1426	63	309	*chinese-roberta-wwm-ext*
BIOSSES	**0.3996**^b^	0.3614^b^	36	17	41	246	*roberta-base*

^a^The value of *Macro F1*, where the bolded one indicates the best performance

^b^The value of *Person correlation*, where the bolded one indicates the best performance

**Table 7:** The experimental results of *transformers-sklearn* and *transformers* in four medical NLP tasks (mode_type = “albert”)

Name	Score		Second		Lines of code		Pre-trained model
	Ours	*Transformers*	Ours	Transformers	Ours	*Transformers*	
TrialClassification	**0.7142**^a^	0.4504^a^	1062	1068	38	246	*albert_chinese_base*
BC5CDR	0.8422^a^	**0.8523**^a^	444	492	41	309	*albert-base-v2*
DiabetesNER	0.6196^a^	**0.6436**^a^	1122	1253	63	309	*albert_chinese_base*
BIOSSES	0.1892^b^	**0.4394**^b^	12	11	41	246	*albert-base-v2*

^a^The value of *Macro F1*, where the bolded indicates the best performance

^b^The value of *Person correlation*, where the bolded inidcates the best performance

**Fig. 2:**
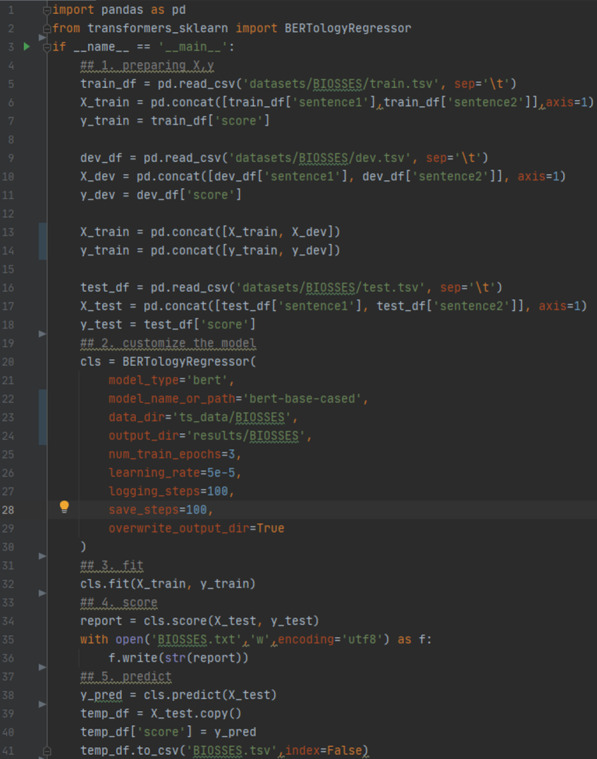
The code for *BIOSSES* within *transformers-sklearn*

As shown in Fig. 2, the entire code for *BIOSSES* implement is short and easy to use. The users could apply *transformer-based* models in the *scikit-learn* coding style with the help of our toolkit. In the four tasks, the average code load of our toolkit’s script is 45 lines/task, which is one-sixth the size of *transformers*’ script. In addition to the comparison of the number of lines of code, we also compared the running time of each model, as shown in Tables 5, 6, and 7, which indicated the high efficiency of *transformers-sklearn*.

## Discussion

### Principal results

The proposed toolkit, *transformers-sklearn*, was proved to be easy to use for newcomers and could be used for *transformer-based* models as the *scikit-learn* coding style.

### Limitations

Compared with *transformers*, the limitation of *transformers-sklearn* is its lack of flexibility. For example, within *transformers-sklearn*, it is impossible for users to extract any encoding or decoding layer of the *transformer*. In other words, users cannot determine which layer of *transformer* could act in the downstream tasks.

Furthermore, *transformers-sklearn* aims at making *transformer-based* models for easy use and expanding the capability of *scikit-learn* in deep learning methods. For advanced users, the *transformers* toolkit is better than our *transformers-sklearn* regarding flexibility.

### Comparison to existing tools

Compared with prior toolkits, such as *transformers* and *UER*, *transformers-sklearn* is easy to get started using for newcomers with basic machine learning knowledge. The experimental results of the four medical NLP tasks showed that the BERT model in *transformers-sklearn* obtained preferable performance while using much less code and comparable running time.

*transformers-sklearn* is based on *transformers*. We wrapped the powerful functions implemented by *transformers* and made them transparent to users. *transformers-sklearn* is also based on *scikit-learn*, which is popularly used in machine learning fields. Thus, the technique advantages of both *scikit-learn* and *transformers* were integrated in our toolkit*.*

## Conclusions

In this paper, three Python classes including *BERTologyClassifier*, *BERTologyNERClassifier* and *BERTologyRegressor* and three methods of each class were developed in *transformers-sklearn*. To validate the effectiveness of *transformers-sklearn*, we applied the toolkit in four multilingual medical NLP tasks. The results showed that *transformers-sklearn* could effectively address the NLP problems in both Chinese and English if the pre-trained *transformer*-based model supported the language. The code and tutorials of *transformers-sklearn* are available at https://doi.org/10.5281/zenodo.4453803.

In future work, a keep-updating *transformers_sklearn* toolkit that combines flexibility and usability will be released, with supporting a wide range of medical language understanding tasks.

### Availability and requirements

The datasets and software supporting the results of this article are available in the trueto/transformers_sklearn repository.

- Project name: transformers-sklearn
- Project home page: https://doi.org/10.5281/zenodo.4453803
- Operating system(s): Windows/Linux/Mac OS
- Programming language: Python
- Other requirements: PyTorch
- License: Apache License 2.0

## Data Availability

The datasets and software supporting the results of this article are available in the trueto/transformer-sklearn repository, https://doi.org/10.5281/zenodo.4453803.

## Abbreviation

BERT: Bidirectional Encoder Representations from Transformers
